# Correction to “Surgical correction of sunken upper eyelid with upper arcus marginalis release and precision fat distribution technique”

**DOI:** 10.1111/jocd.16483

**Published:** 2024-07-22

Hong S‐H, Choi N, Baek R‐M, Kim J‐H. Surgical correction of sunken upper eyelid with upper arcusmarginalis release and precision fat distribution technique. J Cosmet Dermatol. 2024;23:1771–1776

Adding affiliation (Department of Plastic and Reconstructive Surgery, Seoul National University College of Medicine, Seoul National University Bundang Hospital, Seongnam, Korea) for the first author (Sa‐Hyeok Hong).

